# Surgery first – indications, protocols, results

**DOI:** 10.1186/1746-160X-10-S1-O1

**Published:** 2014-12-12

**Authors:** Konrad Wangerin

**Affiliations:** 1 Paracelsus-Krankenhaus Ruit - Klinik für Gesichts-, Kiefer- und Wiederherstellungschirurgie, Ostfildern, Germany

## 

The multi-step treatment of severe dentofacial deformities starts after the termination of skeletal bone growth. In cases of additional transversal discrepancies the SARPE procedure has to be executed at first, followed by the orthodontic alignment of the dental arches. Then the at least three step treatment starts: it consists of the pre- and postoperative orthodontic treatment and the treatment in between surgical correction. This combined treatment may last up to three years and longer.

For economic reasons as well as for an increasing motivation of the patient the ‘Surgery First’ concept was developed to reduce treatment time. We take advantage of the Regional Acceleration Procedure (RAP), which is known since Köle published the interdental corticotomy in the crowded lower front to accelerate the orthodontic alignment in the 1950s.

Instead of hyrax screws for transversal widening we prefer he application of bone borne devices for SARPE procedures. Fixed appliances treatment starts earlier using RAP effect.

Instead of preoperative orthodontic treatment we start with the operation and align the dental arches postoperatively within 6-8 months.

Advantages of ‘Surgery First’:

• The patient sees the operative results immediately.

• Orthodontic treatment is accelerated by RAP effect.

• Treatment is completed after 6-8 months, sometimes after 10 months.

Disadvantages of ‘Surgery First’:

• Orthodontists must be experienced and needs to plan the treatment in advance.

• More close cooperation between surgeon and orthodontist is needed.

• Orthodontic treatment starts 1-2 weeks after the operation with intervals of two weeks.

• RAP effect takes three to four months.

The ‘Surgery First’ concept can be performed easily with stable results in Angle Class II and III cases with crowding, frontal compensation and pronounced Spee curve. (Segmented) Bimaxillary osteotomies with or without CWR or CCWR can be performed with additional procedures as chin correction or jaw bone augmentation.

The ‘Surgery First’ concept delivers safe results also in open bite cases, elongated front regions, minor transversal discrepancies and asymmetries. In cases of severe facial deformities and transversal discrepancies ‘Surgery First’ cannot be recommended yet.

